# Egg-Derived Lipids and Phospholipids as Emerging Functional Ingredients: Molecular Structure, Bioavailability, Metabolic Effects, and Food Applications

**DOI:** 10.3390/foods15193564

**Published:** 2026-10-08

**Authors:** Osama K. Abou-Emera, Mohammed Aladhadh

**Affiliations:** 1 Department of Animal and Poultry Production, College of Agriculture and Food, Qassim University, Buraydah 51452, Saudi Arabia; 2 Department of Poultry Breeding Research, Animal Production Research Institute, Agriculture Research Centre, Dokki, Giza 12618, Egypt; 3 Department of Food Science and Human Nutrition, College of Agriculture and Food, Qassim University, Buraydah 51452, Saudi Arabia; aladhadh@qu.edu.sa

**Keywords:** egg-yolk phospholipids, phosphatidylcholine, lipoprotein fractionation, lipid bioaccessibility, choline bioavailability, supercritical fluid extraction, liposomal delivery, functional food ingredients

## Abstract

Egg yolk is often treated in the functional-food literature as a single, compositionally uniform fat depot, but this simplification obscures its actual structure: the yolk is a compartmentalized lipid system in which egg-yolk lipids are distributed between a triglyceride-dominant, low-density-lipoprotein (LDL) plasma fraction and a phospholipid-enriched, high-density-lipoprotein (HDL) granule fraction. Prior reviews of egg-yolk lipid bioactivity, extraction methodology, and flavor chemistry have generally addressed these topics as separate studies; here, molecular architecture, extraction and characterization, gastrointestinal digestion and bioaccessibility, human bioavailability and metabolic fate, health outcomes, and technological functionality are synthesized as stages of a single pathway. The compositional asymmetry between the LDL plasma fraction and the HDL granule fraction is well established, and differences in their emulsifying behavior are supported by the technological literature; divergent metabolic effects of the isolated fractions have so far been shown only in animal models, whereas differences in digestion kinetics are inferred from structure and remain a working hypothesis because direct experimental comparison of the digestion of isolated HDL and LDL fractions is still lacking. On the human evidence side, a single randomized crossover trial in 18 healthy adults found a higher plasma choline response to phospholipid-bound choline than to choline delivered as a synthetic salt, a preliminary finding that awaits replication, while two crossover studies found no significant postprandial rise in plasma cholesterol area under the curve after whole-egg intake. Because this is an indirect, postprandial measure rather than a direct assessment of intestinal absorption, it is consistent with, but does not establish, limited cholesterol absorption from the whole-egg matrix, and whether whole-egg cholesterol should be distinguished from isolated dietary cholesterol exposure in the epidemiological literature remains a hypothesis. Large prospective meta-analyses report that egg and dietary cholesterol intake associate modestly with elevated all-cause and cancer mortality, but not with cardiovascular-disease-specific mortality or events; these observational associations describe health outcomes and do not, on their own, test a mechanistic distinction between whole-egg cholesterol and isolated dietary cholesterol absorption. Extraction and delivery-system technology for egg phospholipids is more advanced than commonly assumed, with pilot-scale supercritical-fluid purification already achieved and egg-yolk phosphatidylcholine reported to show formulation-specific advantages over soybean phosphatidylcholine in individual liposome studies, although these studies differ in cargo, preparation method, and outcome measures. This delivery-system evidence, however, remains at the level of isolated liposome formulations; finished food products incorporating egg-phospholipid liposomes, with accompanying sensory or consumer-acceptance validation, have not yet been reported. The principal barriers to validated functional-ingredient status are correspondingly narrower than a general shortfall in bioactivity or functionality evidence: a direct human bioavailability comparison between whole egg and an isolated egg-phospholipid ingredient, and dedicated techno-economic and life-cycle assessment of egg-lipid extraction, remain the two outstanding gaps.

## 1. Introduction

Few foods carry as much nutritional density packed into as little structural complexity as the hen’s egg, and within the egg, the yolk is where that complexity concentrates. Roughly a third of whole-yolk mass is lipid, and although triacylglycerols dominate that fraction by weight, egg yolk remains the richest natural source of phospholipids and cholesterol among foods commonly consumed. This lipid fraction does not exist as a bulk oil phase but is embedded within discrete high- and low-density lipoprotein particles and granules, with phosphatidylcholine as the dominant phospholipid class, trailed by phosphatidylethanolamine, sphingomyelin, plasmalogens, and phosphatidylinositol [1]. This organized lipoprotein architecture, rather than an undifferentiated fat pool, forms the structural premise underlying most of the mechanistic arguments developed in this review.

This structural organization has attracted growing attention as the search for natural, food-derived phospholipid sources for emulsification, encapsulation, and nutraceutical delivery has intensified. Egg-yolk lipid fractions, including egg-yolk oil, phospholipids, and constituent fatty acids, have been reported to carry anti-inflammatory, antioxidant, cardiovascular-protective, and memory-related bioactivities tied to the regulation of cell function and physiological homeostasis [1]. Commercial uptake of egg-yolk lipids nonetheless remains modest, despite extraction routes (solvent-based and supercritical-fluid-based alike) that are comparatively straightforward by the standards of natural-product isolation [2]. This gap does not appear to reflect a shortage of demonstrated laboratory-scale bioactivity or functionality; rather, it reflects a shortage of translational evidence needed to move from laboratory demonstration to validated, scaled functional-ingredient status.

Part of the reason that gap persists is that the existing review literature has addressed pieces of this pathway in relative isolation, rather than as a connected whole. Xiao et al. [1] compiled bioactivity evidence for egg-yolk oil, phospholipids, and fatty acids, but stopped short of integrating digestion, bioaccessibility, or bioavailability as a mechanistic bridge to that bioactivity, and did not extend into extraction or food-application territory. Abeyrathne et al. [2] offered a detailed comparison of separation and extraction methodologies, yet treated downstream “utilization” narrowly, without a digestion–absorption–metabolism synthesis, and predates most of the 2023–2026 lipidomics and delivery-system literature this review draws on. Chen et al. [3] took on a comprehensive treatment of egg-yolk phospholipids as precursors of fatty-note odorants, a narrow, single-topic scope that this review deliberately does not attempt to reproduce. A 2026 review on whole-egg nutrient-delivery and fortification strategies [4] sits adjacent to the present scope but addresses something distinct: enrichment of the intact egg, typically via hen diet, as a nutrient-delivery vehicle, rather than the extraction, characterization, and independent functional use of egg-derived lipids in other food systems. None of these four reviews traces the full pathway from isolated lipid or phospholipid structure through digestion, bioaccessibility, and metabolism to validated technological functionality in food systems beyond the egg itself, and providing that integration is the specific aim of the present review.

Accordingly, the objective here is to synthesize evidence spanning egg-lipid molecular architecture, extraction and characterization, digestion and bioavailability, metabolic and health effects (including a balanced treatment of dietary cholesterol), and technological and food-application functionality. At each step, in vitro, animal, observational, and human intervention data are treated as distinct tiers of evidence rather than pooled into a single claim, since these data types are not interchangeable in the confidence they support. This distinction is maintained throughout not for its own sake, but because it clarifies what is actually known and, in turn, which specific research is still required to move egg-derived lipids from documented bioactive compounds to validated functional ingredients.

### Literature Search Strategy

This review follows a narrative rather than a systematic design. The relevant literature was identified through searches of PubMed, Scopus, and Web of Science, covering publications from January 2015 to September 2026 (earlier foundational studies identified through hand-searching were also included), with an emphasis on studies published from 2023 onward to capture recent lipidomic and delivery-system advances. Search terms combined “egg yolk,” “egg phospholipid,” “phosphatidylcholine,” “lipoprotein,” “LDL,” “HDL,” “choline bioavailability,” “cholesterol absorption,” “liposome,” and “egg lecithin” with terms specific to each thematic section (e.g., “extraction,” “digestion,” “bioaccessibility,” “mortality,” “emulsification”). Reference lists of retrieved articles and existing reviews [1,2,3,4] were hand-searched for additional relevant studies. Only English-language, peer-reviewed publications were considered. In vitro, animal, observational, and human-intervention studies were classified separately and are labeled by evidence tier throughout the review (see Section 7, Section 8, Section 9 and Section 10). Statements that “no study was identified” reflect the outcome of this search strategy within the stated databases, time window, and language restriction, and should be read as a description of the literature retrieved rather than an exhaustive systematic assessment.

## 2. Molecular Architecture of Egg-Derived Lipids

### 2.1. Overall Lipid Composition

Egg yolk is approximately 33% lipid by wet weight. Within that lipid fraction, three major classes account for essentially all of the mass: triacylglycerols, phospholipids, and cholesterol, though the exact proportions reported vary across studies and analytical methods. Compositional analyses converge on a yolk lipid fraction of roughly 62–70% triacylglycerol, 25–33% phospholipid, and 4–5% cholesterol, with minor amounts of other lipids making up the remainder [5,6]. The spread in these figures more likely reflects breed, diet, and analytical-method variation than genuine biological disagreement, a point this review returns to in Section 16 as a standardization gap.

### 2.2. Lipoprotein Organization

What matters functionally about egg-yolk lipids is not their overall composition but their physical location. Egg yolk separates into an insoluble granule fraction and a continuous aqueous plasma fraction, and these two fractions differ sharply in both bulk composition and lipid organization. Yolk plasma is lipid-dominant (approximately 70% lipid against 22% protein) and its lipids exist mainly as low-density lipoprotein (LDL). Granules run the opposite way: protein-dominant at roughly 61% protein against 19% lipid, with their lipid component existing mainly as phospholipid-rich high-density lipoprotein (HDL) [5,6]. Zooming in further, yolk plasma itself comprises approximately 85% LDL and 15% globular glycoproteins (livetins), while granules consist of approximately 70% HDL, 16% phosvitin, and 12% LDL. At the level of the individual particle, LDL carries an inner core of triglycerides and cholesteryl esters wrapped in a surface layer of phospholipids, cholesterol, and apoproteins; lipid accounts for 83–89% of particle weight, split roughly 69% triglycerides, 26% phospholipids, and 5% cholesterol [6].

The practical result is a structural inversion: HDL/granule particles are phospholipid-enriched relative to the triglyceride-dominant LDL/plasma particles. Digestion kinetics, emulsification behavior, and lipase accessibility are expected to differ between these two particle populations, not merely between lipid classes considered abstractly but between the physical compartments those classes actually occupy in the yolk; direct experimental confirmation of divergent digestion kinetics between isolated HDL and LDL fractions is, however, still lacking (Section 16, Gap 7), so this compartmentalization is used throughout the review as a working explanatory hypothesis rather than an established finding.

### 2.3. Phospholipid Classes

Phosphatidylcholine (PC) is, by every analytical platform used to date, the dominant egg phospholipid class, though the precise share attributed to it (and the ranking of the minor classes behind it) shifts somewhat depending on method. Reported values place PC at approximately 72–77% of total phospholipid, phosphatidylethanolamine (PE) at 15–20%, and lysophosphatidylcholine, phosphatidylinositol, sphingomyelin, and phosphatidylserine each in the low single digits [3,6]. Mass-spectrometry-based structural characterization has resolved the major species within these classes, including PC(16:0–18:1), PE(18:0–20:4), PI(18:0–18:2), PS(18:0–18:2), SM(d18:1/16:0), and LPC(16:0) [6].

This classical picture, however, has recently proven to be an undercount. High-resolution untargeted lipidomics has expanded the known phospholipid diversity of egg yolk considerably: one recent characterization identified 19 phospholipid classes comprising 275 molecular species and reported for the first time that egg yolk is also rich in glucosylceramides, galactosylceramides, lactosylceramides, gangliosides, and plasmalogens carrying polyunsaturated fatty acids (PUFAs) at the sn-2 position [7]. The position along the glycerol backbone at which a PUFA is esterified (in glycerophospholipids, the fatty acyl chains are esterified at the sn-1 and sn-2 positions of the glycerol backbone, whereas the sn-3 position carries the phosphate-linked polar head group) influences a phospholipid’s in vivo bioavailability and function considerably. In egg yolk, docosahexaenoic acid, eicosapentaenoic acid, α-linolenic acid, and arachidonic acid were found esterified at the sn-1 position of PC and at the sn-2 position of several PE species [7], a positional pattern relevant again when digestion and absorption are discussed in Section 6 and Section 7.

### 2.4. Fatty-Acid Composition

Across studies, the major yolk fatty acids by weight are oleic acid (C18:1 n-9, roughly 30–44%), palmitic acid (C16:0, roughly 22–54%), linoleic acid (C18:2 n-6, roughly 0.4–17%), and stearic acid (C18:0, roughly 4–12%) [8]. The width of these ranges reflects how strongly the fatty-acid profile responds to hen diet and breed, which is relevant to any downstream ingredient-standardization effort.

### 2.5. Structural Determinants of Functionality

**Figure 1:**
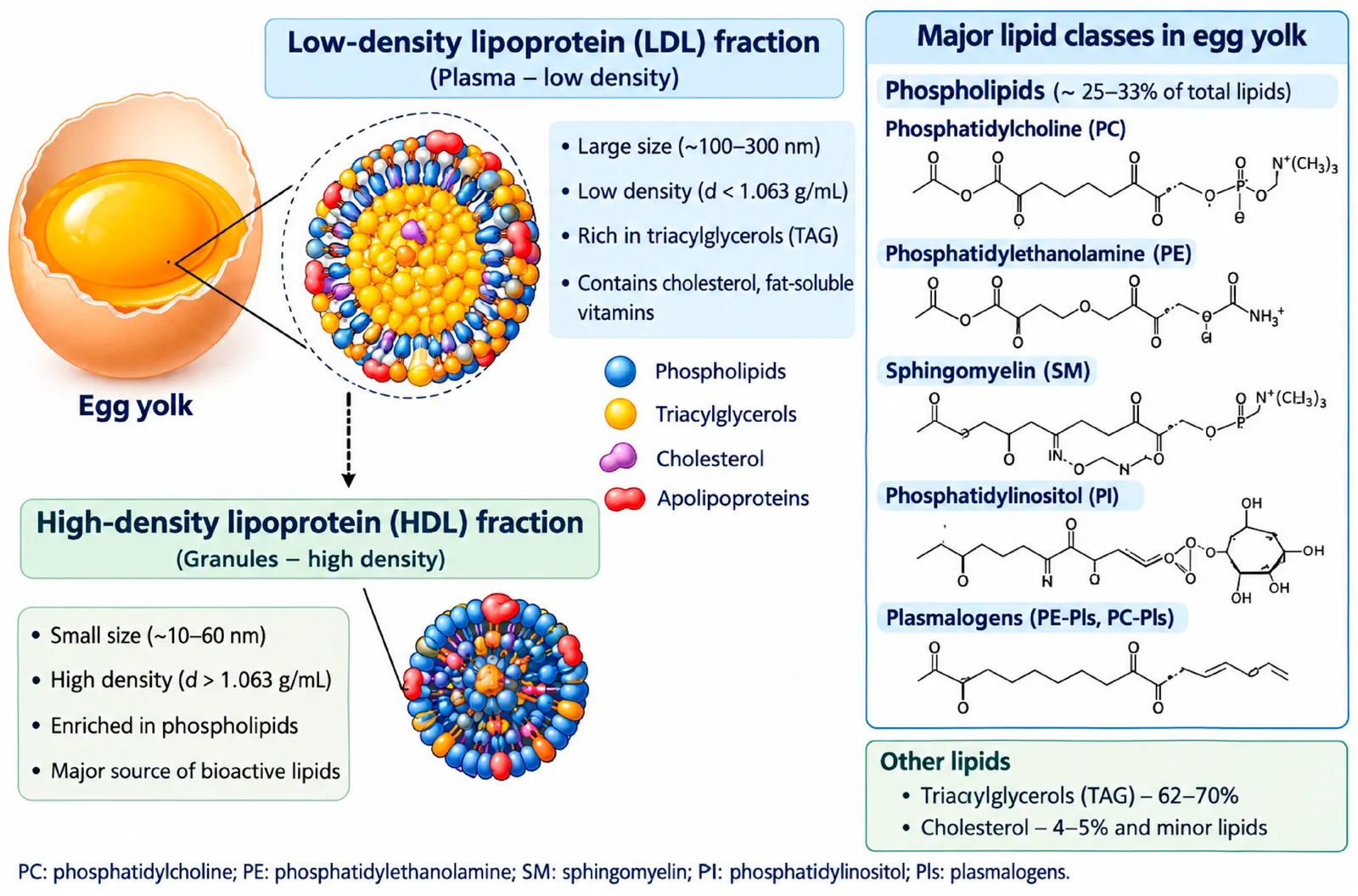
Molecular architecture of egg-yolk lipids. The yolk’s total lipid content is compartmentalized asymmetrically between a triglyceride-dominant LDL/plasma fraction and a phospholipid-enriched HDL/granule fraction.

Three structural facts established in this section recur throughout the review as explanatory variables. First, egg phospholipids are compartmentalized asymmetrically between a triglyceride-rich LDL/plasma pool and a phospholipid-rich HDL/granule pool, which affects lipase accessibility during digestion (Section 6). Second, PC dominance combined with PUFA enrichment at variable sn-positions governs micellar solubilization efficiency and absorption route (Section 7). Third, the newly resolved diversity of sphingolipid and plasmalogen species constitutes a class of minor but potentially bioactive lipids whose functional roles remain largely uncharacterized (Section 16). These three facts together form the structural basis for much of the discussion that follows. These figures are summarized in Table 1. The molecular architecture of egg-yolk lipids is illustrated in Figure 1.

The corresponding phospholipid-class distribution is summarized in Table 2.

## 3. Extraction, Fractionation, and Purification

A direct consequence of the structural picture established in Section 2 is that egg-yolk lipids resist simple extraction, since phospholipids are bound tightly to protein and cholesterol within the lipoprotein architecture described above. This tight association is why egg and dairy phospholipids generally require more complex, multi-step extraction procedures than plant-derived phospholipids, which can often be recovered by comparatively simple degumming or solvent fractionation [9].

Against that backdrop, conventional solvent extraction (the Folch and Bligh and Dyer methods) remains the benchmark for total lipid and phospholipid recovery. Folch tends to give the best recovery from lipid-rich matrices such as egg yolk, while Bligh and Dyer performs comparatively better in low-fat, high-moisture matrices; both, however, under-recover minor acidic phospholipids and rely on chloroform, which is neither environmentally benign nor pleasant to work with at scale [9]. Supercritical carbon dioxide (SC-CO_2_) extraction is the most extensively studied “green” alternative specifically evaluated for egg yolk. An early comparative study found SC-CO_2_ yielded 67 g extract per 100 g sample, against 63 g/100 g for conventional Bligh and Dyer extraction, yet the solvent extract actually contained more phospholipid (29%) than the SC-CO_2_ extract (26%) [10]. This inversion arises because phospholipids are amphiphilic and poorly soluble in neat CO_2_, so efficient extraction generally requires either elevated pressure or a polar cosolvent [9,10].

Moving from bench-scale extraction to purification, pilot-scale isolation combining liquid-ethanol or SC-CO_2_ extraction with a supercritical-antisolvent precipitation step has achieved phosphatidylcholine purity of approximately 87% from fresh yolk and 84% from cream at 20% moisture content, with the resulting precipitate 72–99% PC plus PE, in ratios of 6:1 to 7:1 depending on process conditions [11]. A separate line of work applied SC-CO_2_ extraction with ethanol cosolvent to leftover yolk following immunoglobulin Y (IgY) extraction (a useful byproduct-valorization scenario) and achieved PC recovery/purity of approximately 94%/57% from enzymatically treated cream, against 47%/47% from dry yolk and 40%/75% from pellet. Protease pretreatment of the pellet raised phospholipid recovery to 86% (from 49% untreated) but simultaneously dropped purity to 67% [12]. This is worth stating plainly, because it is easy to read past: protease pretreatment does not improve recovery and purity together. It trades one for the other, and moisture content behaves the same way: the highest PC and total-phospholipid recoveries generally occur for freeze-dried or low-moisture cream, while the highest PC purity is reported for fresh, undried yolk [12].

A direct three-way comparison of extraction methods indicates that method choice determines which lipid class is favored, not simply how much total material is recovered. SC-CO_2_ extraction (supercritical fluid extraction, SFE) yields extracts high in unsaturated fatty acids with low acid and peroxide values but largely leaves polar phospholipids behind in the yolk powder rather than extracting them. Subcritical propane extraction, by contrast, achieves the highest overall lipid extraction efficiency of the three methods compared, with phospholipid extraction capacity falling intermediate between SFE and conventional solvent extraction [13]. Two further green methods, switchable-hydrophilicity solvents and natural deep eutectic solvents, have been applied across dairy, marine, and egg matrices with reduced solvent consumption, but egg-specific recovery and purity benchmarks for these newer approaches remain thin in the literature identified for this review [9]. The yield, purity, and limitations of the extraction and fractionation technologies discussed above are compared in Table 3.

## 4. Advanced Characterization of Egg Lipids

**Figure 2:**
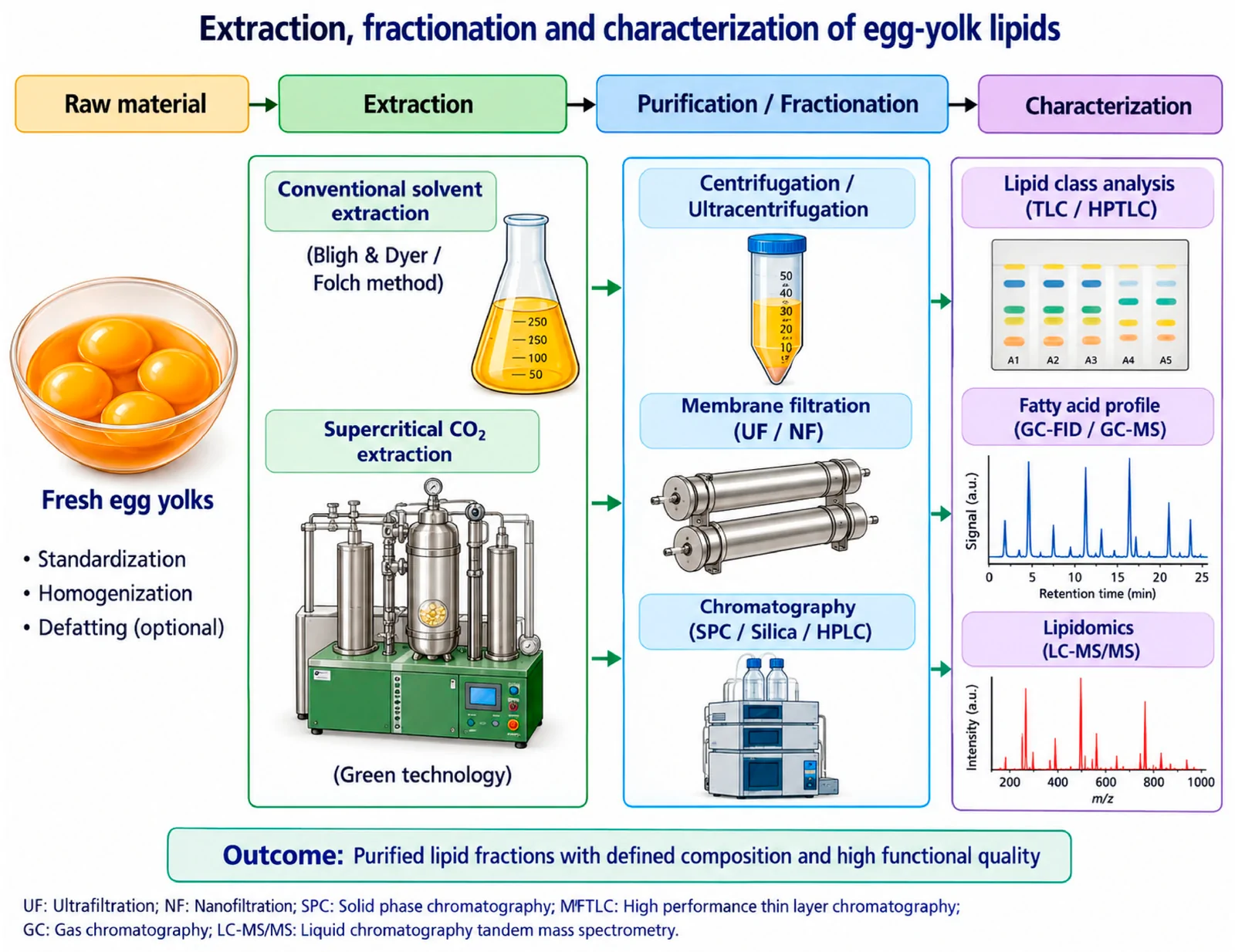
Extraction, fractionation, and characterization workflow for egg-yolk phospholipids. Method choice determines which lipid class is preferentially recovered rather than simply overall yield; no single characterization technique is sufficient alone, motivating the orthogonal, multi-method approach shown.

Characterizing what has been extracted is a separate problem from extracting it, and no single analytical technique solves it alone. Thin-layer chromatography (TLC) remains useful for quick qualitative screening. UHPLC/HPLC coupled to universal detectors (CAD/ELSD) is the current workhorse for class-level quantification. LC-ESI-MS(/MS) is indispensable for molecular-species identification, oxidation tracking, and detection of lyso-lipid species. ^31^P NMR offers non-destructive, inherently quantitative class profiling without requiring external calibration standards. GC-FAME delivers high-precision fatty-acyl profiling of already-separated fractions. And untargeted lipidomics is the method responsible for expanding the known egg-yolk phospholipid inventory to 19 classes and 275 molecular species, and for first resolving the sn-position-specific PUFA esterification patterns discussed in Section 2 that bear directly on bioavailability [7,9]. Given that no method covers the full analytical need on its own, the most defensible current approach pairs TLC or UHPLC-CAD/ELSD for class-level quantification with LC-MS/MS or ^31^P NMR for structural resolution or independent quantitative cross-validation. Table 4 lists the strengths and limitations of each technique. Figure 2 outlines the overall extraction, fractionation, and characterization workflow.

## 5. Processing, Oxidation, and Structural Stability

Egg-yolk phospholipids are structurally labile during processing, with consequences for food quality. Spray-drying causes lipid oxidation, particularly of free fatty acids, together with potential phospholipid hydrolysis and alteration of polyunsaturated fatty acid forms. The corresponding compositional signature is consistent: PA, PC, and PE decrease while the lysophospholipids LPA, LPC, and LPE increase, a pattern attributed to heat-induced structural change in the protein–lipid complex that renders fatty acids more susceptible to liberation, oxidation, and hydrolysis. These alterations are not confined to the drying step itself but are further aggravated during subsequent accelerated storage [14]. Comparative storage work quantifies the scale of the effect: lipid hydroperoxide and thiobarbituric acid values reached up to ten times higher in spray-dried eggs than in fresh eggs after storage. Omega-3-enriched eggs proved more susceptible to oxidation than omega-6-enriched eggs, while α-tocopherol supplementation improved lipid stability throughout storage [14].

Heating intensity matters as much as heating method. A quantitative lipidomic comparison across boiling intensities, spanning 767 quantified lipids, found 190 showing differential abundance across heating intensities, with heat treatment altering lipoprotein assembly structure through thermal denaturation and increasing the abundance of low-to-medium-abundance triglycerides [15]. Decreased phospholipid content alongside increased lysophospholipid and free-fatty-acid content has also been observed in hot-spring and soft-boiled yolk, indicating that phospholipid degradation is not confined to industrial processing such as spray-drying or aggressive heat treatment but occurs at ordinary culinary cooking temperatures as well [15].

Not every oxidation-linked change in this system is undesirable, however. Egg-yolk phosphatidylcholine and phosphatidylethanolamine accelerate methyl linoleate autoxidation under mild conditions, yet the same phospholipids, once collected after high-temperature heating, instead inhibit hydroperoxide formation at the initial oxidation stage, an effect attributed to Maillard browning products generated during heating. The oxidative behavior of egg phospholipids therefore appears to be condition- and compound-dependent rather than a fixed class property, and claims about whether it is beneficial or harmful require specifying temperature, concentration, and reaction context.

## 6. Gastrointestinal Digestion and Bioaccessibility

Before evaluating what egg lipids do once absorbed, three terms that are frequently used interchangeably in this literature should be distinguished: bioaccessibility (the fraction released from the food matrix and solubilized in a form available for absorption), absorption (uptake across the intestinal epithelium), and bioavailability (the fraction that reaches systemic circulation and is available for physiological use). The evidence at each stage tells a somewhat different story.

At the bioaccessibility stage, a multi-compartment in vitro gastrointestinal system was used to test how thermal processing shapes lipid release. Cumulative free-fatty-acid bioaccessibility fell from 25–29% in raw yolk to 20–24% in yolk cooked to 85 °C, with induction time lengthening correspondingly, and bioaccessibility correlated negatively with storage modulus and protein intermolecular β-sheet content while correlating positively with peak enthalpy and native protein content [16]. The same protein network that gives cooked yolk its texture appears to govern how much lipid becomes accessible to digestion, consistent with the protein–lipid matrix structure described in Section 2, and in this system thermal processing reduces rather than increases lipid bioaccessibility.

The granule vs. plasma distinction from Section 2 also appears at the digestion stage. Using the standardized INFOGEST 2.0 protocol, high-hydrostatic-pressure (HHP) treatment of egg yolk and granule altered lipid bilayer/micelle formation and contributed to higher phospholipid content detected at the oral phase and after 60 min of gastric digestion in HHP-treated granule relative to untreated control. HHP left overall protein digestibility unchanged but increased the susceptibility of phosvitin to proteolysis, generating bioactive phosphopeptides in the process [17]. A separate in vitro human digestion model examined whether the food matrix itself could be engineered to slow lipid digestion. Biopolymer encapsulation of egg yolk (with pectin, chitosan, or cellulose) reduced the digestion rate of total lipids and cholesterol relative to unencapsulated yolk, and pectin- and chitosan-encapsulated samples specifically showed lower free-fatty-acid content, lower lipid-oxidation values, and decreased lipase activity after digestion [18]. This provides direct evidence that matrix modification is a viable lever for controlling egg-lipid digestibility, a point taken up again when delivery-system design is discussed in Section 12.

## 7. Bioavailability and Food-Matrix Effects

Section 6 addresses how much lipid becomes accessible to digestion; this section considers what happens once digestion is complete. Somewhat unexpectedly, the strongest human evidence available concerns not a lipid but choline. In a randomized, double-blind, crossover trial in 18 healthy adults, plasma choline response (incremental area under the curve, AUC) was four times higher after consumption of an egg-yolk phospholipid drink (3 g phospholipid-bound choline) than after an equivalent 3 g of choline delivered as choline bitartrate salt, indicating improved bioavailability of naturally phospholipid-bound choline relative to choline-salt intake, though the precise mechanism behind that difference remains a matter of speculation rather than established fact [19]. Because this finding derives from a single crossover trial in 18 healthy adults, it should be regarded as a preliminary rather than definitive demonstration of the bioavailability advantage, pending replication in a larger and more diverse population. A separate trial in adults with metabolic syndrome adds a real-world data point: intake of three eggs per day, as a phosphatidylcholine source, and an equivalent choline-bitartrate supplement both raised plasma choline over four weeks, consistent with egg-derived choline being well absorbed, without a significant change in plasma trimethylamine-N-oxide (TMAO) levels [20].

Cholesterol behavior differs in ways that carry more consequence for the mortality data discussed later. Two randomized controlled crossover studies found that co-consuming cooked whole egg (75–150 g) did not significantly change plasma total-cholesterol area-under-the-curve in triacylglycerol-rich lipoprotein fractions relative to no-egg controls, even though triacylglycerol AUC did increase significantly in the same experiments. The authors interpreted this as indicating that dietary cholesterol in whole egg is not well absorbed [21]. Postprandial plasma cholesterol AUC, however, is an indirect, downstream marker rather than a direct measure of intestinal cholesterol absorption, and the comparison was made against no-egg controls rather than against isolated cholesterol; the findings are therefore suggestive evidence that the whole-egg food matrix may limit the postprandial plasma appearance of cholesterol, not direct demonstration of reduced absorption. This interpretation rests on postprandial area-under-the-curve measurements taken over a single meal in two crossover studies, rather than on direct absorption measures or on longer-term absorption-balance or clinical-outcome data, and the generalizability of the finding beyond the tested dose range (75–150 g cooked egg) has not been established. A structural mechanism has been proposed to explain this: egg-yolk phosphatidylcholine may interact with the intestinal cholesterol transporters NPC1L1 (Niemann-Pick C1-Like 1) and SR-B1 (Scavenger Receptor class B type 1), potentially hindering cholesterol absorption [22]. This mechanism is plausible and consistent with the observed postprandial data but has not yet been directly confirmed in humans.

**Figure 3:**
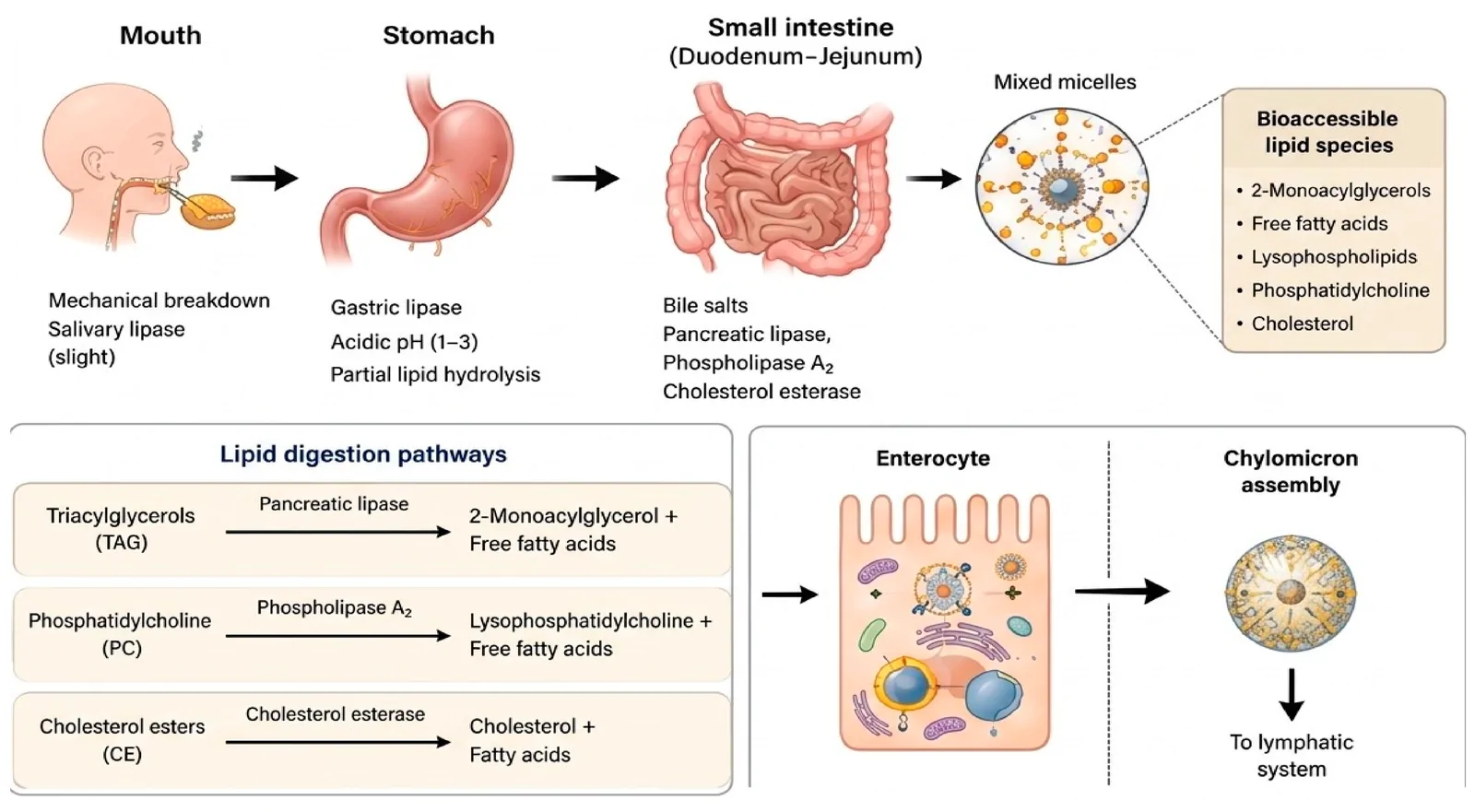
Gastrointestinal digestion, bioaccessibility, absorption, and metabolic fate of egg-derived lipids. Bioaccessibility, absorption, and bioavailability are shown as distinct stages rather than interchangeable terms; the choline/cholesterol branch illustrates that the egg-yolk matrix may favor the plasma appearance of phospholipid-bound choline while limiting that of cholesterol (preliminary evidence).

Together, the choline and cholesterol bioavailability evidence suggests that the egg-yolk food matrix may act as an active modulator of what becomes bioavailable rather than a passive carrier, possibly favoring the plasma appearance of phospholipid-bound choline while limiting that of the co-located neutral cholesterol, although both inferences rest on limited human evidence. Whether these same matrix effects persist when the phospholipid is isolated from the whole egg cannot be answered from the current evidence base. No human intervention study directly comparing whole-egg consumption against isolated or purified egg-phospholipid ingredient consumption was identified in this review, and this absence is flagged in Section 16 as a priority gap, arguably one of the two most consequential ones identified here. The findings discussed above are consolidated in Table 5. Gastrointestinal digestion, bioaccessibility, absorption, and metabolic fate is illustrated in Figure 3.

## 8. Metabolic Fate and Molecular Effects

Most of what is known mechanistically about how egg-yolk lipids affect lipid metabolism comes from animal models, and the strongest of these directly test the structural asymmetry established in Section 2. A direct comparison of egg-yolk LDL vs. HDL fractions fed to mice found that LDL intake increased liver weight and significantly increased plasma and liver cholesterol, while HDL intake decreased body weight and epididymal fat in a dose-dependent manner, with food efficiency decreasing as dietary phospholipid content rose [23]. The same LDL/HDL compartmentalization that is hypothesized to shape digestion kinetics (Section 2.2) also produces sharply divergent metabolic outcomes when the fractions are fed separately, indicating that the structural asymmetry described in Section 2 has functional consequences beyond digestion, even though the digestion-kinetics side of this relationship itself remains experimentally untested (Section 16, Gap 7). Mechanistically, in high-fat-diet-induced obese mice, egg-yolk HDL downregulated hepatic SREBP-1c expression (3.87-fold and 9.43-fold at 60 and 100 days, respectively), with a parallel decrease in Elovl6 expression, indicating suppressed de novo lipogenesis [24]. An in vivo isotope-tracer model adds a further layer, showing that egg-yolk intake induced reciprocal changes in glycerolipid metabolism, increasing hepatic phospholipid secretion into blood while decreasing triacylglycerol secretion [25]. These findings derive entirely from mouse and rat models; species differences in lipoprotein metabolism, bile-acid physiology, and hepatic lipogenic regulation between rodents and humans mean that the magnitude, and in some cases the direction, of these effects cannot be assumed to translate directly to human physiology without dedicated human validation.

**Figure 4:**
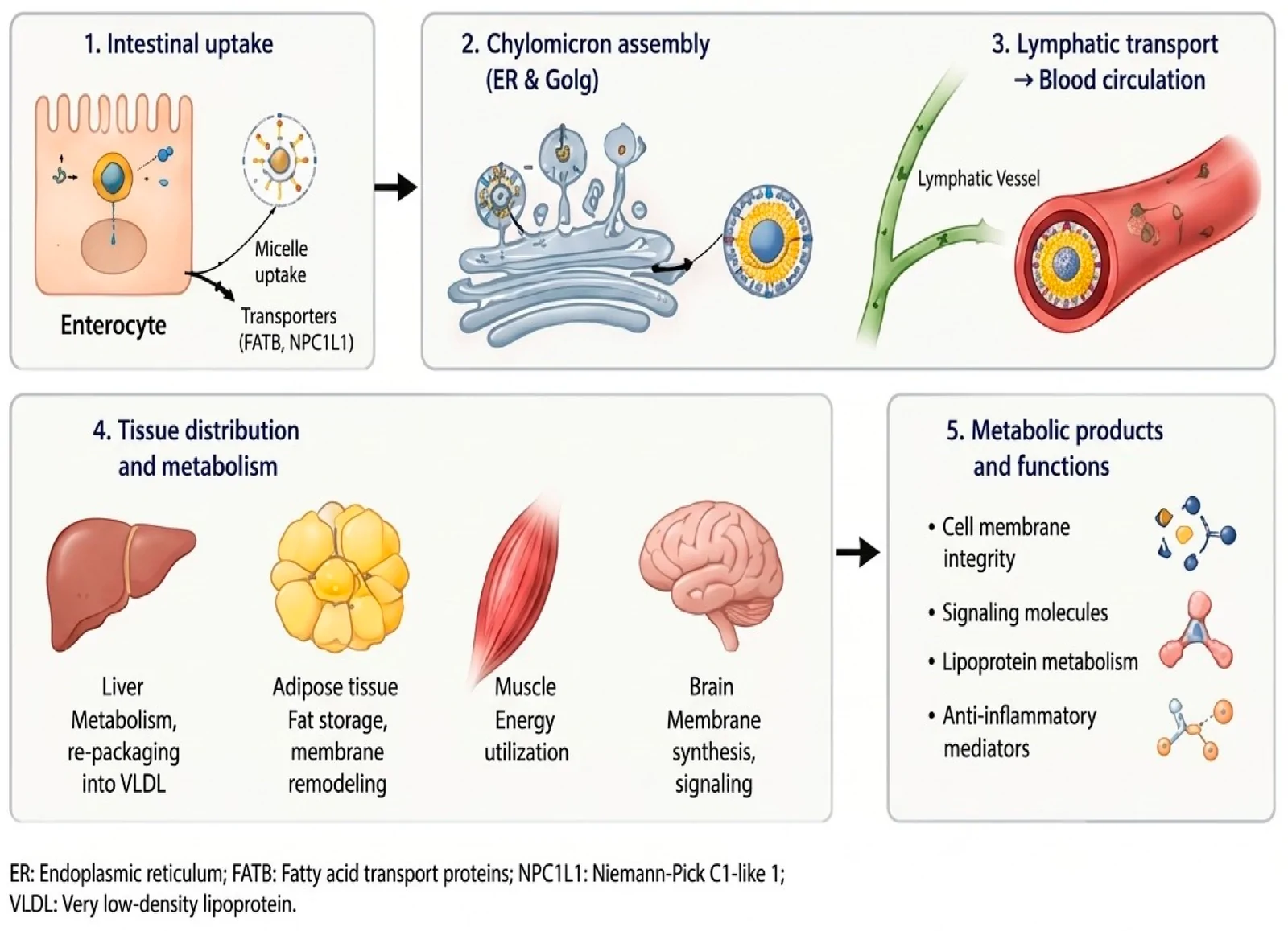
Bioavailability and metabolic fate of egg-yolk phospholipids. The ‘Brain’ and ‘Anti-inflammatory mediators’ outcomes shown here are based on animal or non-egg-specific mechanistic evidence; egg-specific human confirmation of these functions has not yet been reported (Section 9 and Section 16, Gap 8).

These effects are not fixed properties of egg yolk in isolation, however, but depend on dietary context. Egg yolk added to a normal diet did not adversely affect serum triglycerides, cholesterol, or glucose handling in mice, whereas egg yolk added to a high-fat diet markedly improved these same parameters relative to high-fat diet alone [26]. This context-dependence is an important qualifier for translating the evidence to human nutrition claims, since it suggests egg-yolk lipid effects may be more corrective than uniformly beneficial, most pronounced against a background of existing metabolic dysfunction. A complementary line of evidence from high-fructose-fed rats found that both egg-yolk and soybean phospholipids reduced hepatic triglyceride, oxidative stress markers, and inflammatory markers, with egg-yolk phospholipids specifically increasing blood and brain acetylcholine content [27], a source-specific effect that connects lipid metabolism to the cognitive-bioactivity claims examined next. Figure 4 summarizes the bioavailability and metabolic fate of egg-yolk phospholipids.

## 9. Health Effects: Evidence-Based Evaluation

The epidemiological picture for egg and cholesterol intake is more specific, and considerably more consistent, than the binary “eggs are harmful” or “eggs are safe” framing that dominates popular discussion. A dose–response meta-analysis of 55 prospective cohorts, covering more than 2.7 million individuals, found each additional egg per day associated with a 7% higher risk of all-cause mortality and a 13% higher risk of cancer mortality, but no association with cardiovascular mortality; each 100 mg/day increment in dietary cholesterol showed a similar pattern, with elevated all-cause and cancer mortality risk but no CVD mortality association [28]. This CVD-specific null finding is not an isolated result. Three large US prospective cohorts found that consumption of at least one egg per day was not associated with incident cardiovascular disease after adjustment for lifestyle and dietary confounders (HR 0.93, 95% CI 0.82–1.05) [29]. A broader meta-analysis spanning 1966–2020, pooling 23 prospective studies across more than 1.4 million individuals, reached essentially the same conclusion: egg consumption above one egg per day was not associated with a significantly increased risk of overall cardiovascular disease events relative to no or one egg per day, with a pooled HR of 0.99 [30]. An independent dose–response meta-analysis of egg consumption and cardiovascular risk [31], together with a separate umbrella review of systematic reviews and meta-analyses on egg consumption and health outcomes [32], are broadly consistent with this pattern, so the absence of a clear CVD-specific association is a repeated finding across several independently conducted analyses rather than an artifact of any single study design or research group.

It should be noted that these meta-analyses pool cohorts that differ in baseline dietary pattern, egg-preparation method, and population characteristics, and this heterogeneity has not been fully resolved in subgroup analyses; the consistency of the null CVD association across independent analyses strengthens confidence in the finding but does not eliminate cohort-level heterogeneity as a source of residual uncertainty. More broadly, the all-cause- and cancer-mortality associations reported above are observational and cannot establish causation: residual confounding by dietary pattern (e.g., co-occurring processed-meat or refined-carbohydrate intake in high-egg-consumption diets), lifestyle covariates (smoking, physical activity, overall diet quality), and diagnostic or reverse-causation bias in prospective cohorts cannot be fully excluded. These associations should therefore be interpreted as hypothesis-generating rather than causally established, consistent with the evidence-tier distinctions maintained throughout this review.

Cognitive-effect evidence requires a different kind of caution, one concerned less with study quality and more with source attribution. A randomized, double-blind trial found that a marine-sourced phosphatidylserine-omega-3 preparation (PS-DHA) significantly improved verbal immediate recall in non-demented elderly adults with memory complaints [33]. This is genuine evidence of phospholipid-linked cognitive benefit, but it concerns marine-sourced phosphatidylserine, not egg-derived phospholipid, and should not be conflated with egg-specific cognitive bioactivity. No human randomized controlled trial testing isolated egg-derived phosphatidylserine or phosphatidylcholine on cognitive outcomes was identified in this review, a gap addressed further in Section 16.

## 10. Cholesterol: A Critical and Balanced Analysis

Pulling together the absorption evidence from Section 7 and the mortality evidence from Section 9 supports a specific, qualified position, rather than either extreme of “egg cholesterol is harmful” or “egg cholesterol is completely harmless.” Four points anchor that position. First, absorption may be matrix-limited: two randomized crossover studies found no significant postprandial rise in plasma cholesterol AUC after whole-egg intake [21], which is suggestive of limited cholesterol absorption from the whole-egg matrix, although direct absorption measures are lacking (Section 7). Second, endogenous regulation buffers whatever dietary intake does occur, since increased dietary cholesterol is associated with reduced endogenous synthesis via negative feedback on HMG-CoA reductase activity (with genetic factors producing distinct “hyper-responder” and “hypo-responder” subgroups [22]). Third, mortality and cardiovascular-specific outcomes diverge sharply in the evidence: dietary cholesterol and egg intake associate modestly with all-cause and cancer mortality, yet consistently show no association with CVD-specific mortality or events across independent meta-analyses [28,29,30]. Fourth, egg-yolk phosphatidylcholine may specifically offset cholesterol absorption through a proposed interaction with the NPC1L1/SR-B1 transporters [22], though, as noted in Section 7, this mechanism still awaits direct human confirmation.

**Figure 5:**
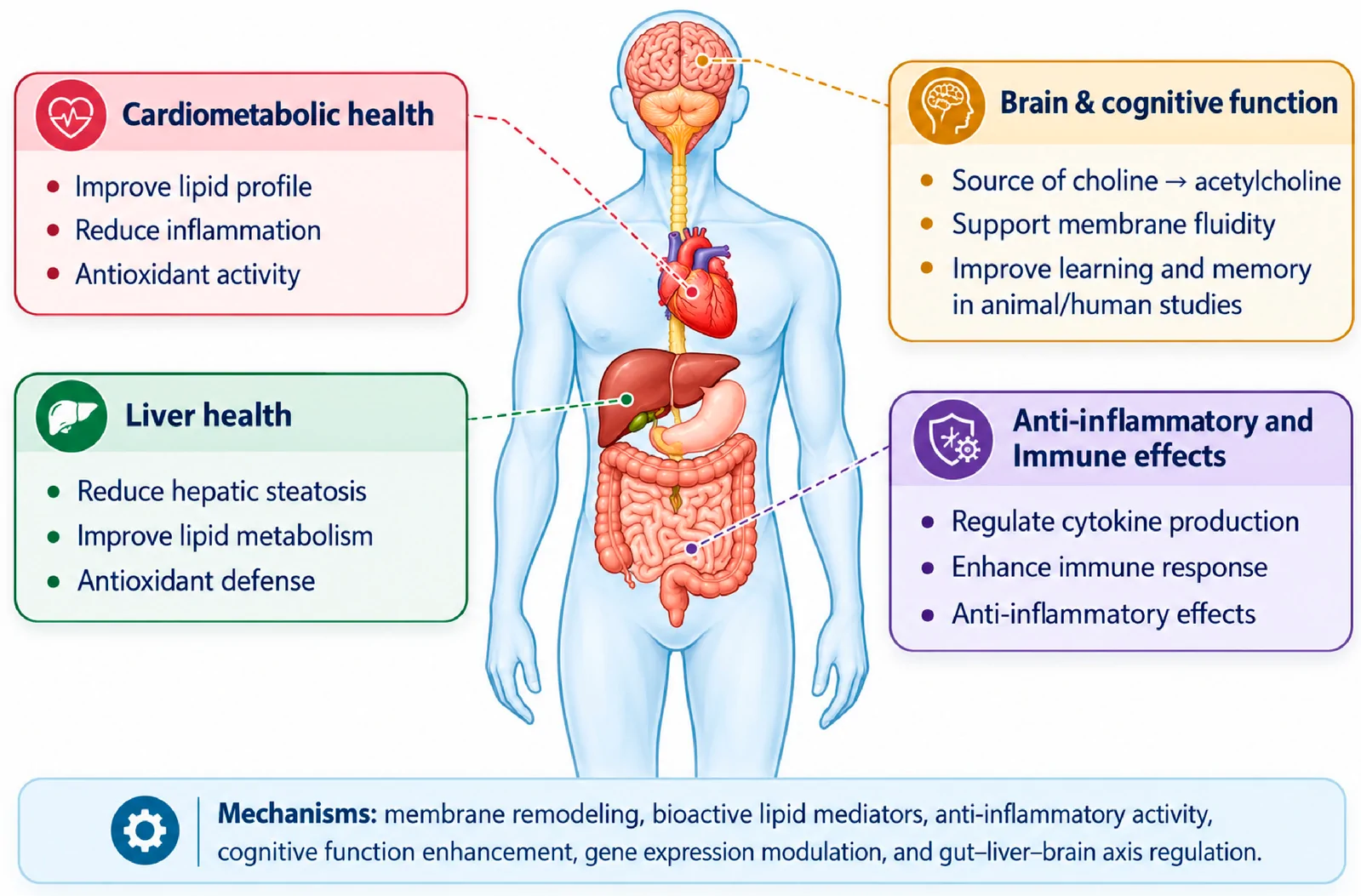
Reported health effects and mechanisms of egg-yolk phospholipids. Cognitive and anti-inflammatory findings shown here derive from marine-phospholipid or animal-model evidence, respectively, not from egg-specific human trials; egg-specific human confirmation remains an identified gap (Section 9 and Section 16, Gap 8). Only outcomes for which egg-specific evidence is discussed in this review are shown. Outcomes for which such evidence was not identified (e.g., gut, skin, eye and bone health, endothelial function, and immune modulation) are not depicted.

These four points converge on a distinction this review treats as important but still provisional. Egg consumption, as a whole-food matrix, has shown no significant postprandial rise in plasma cholesterol AUC in two crossover studies, which is suggestive of limited cholesterol bioavailability, and no clear CVD-specific association, despite modest all-cause and cancer mortality associations whose mechanism remains uncertain. Isolated dietary cholesterol exposure, the metric used in most mortality dose–response analyses, should not be assumed identical to cholesterol delivered within the egg-yolk lipoprotein/phospholipid matrix. And egg-derived phospholipid consumed as an isolated ingredient has, as yet, no identified direct human cardiovascular-outcome data of its own; its cholesterol-related effects are currently inferred from the whole-egg literature rather than independently demonstrated. Collapsing these three categories into a single “eggs and cholesterol” claim, as much popular and even some scientific discourse tends to do, obscures the distinction proposed here. The biological and metabolic evidence discussed in Section 7, Section 8, Section 9 and Section 10 is collected in Table 6. Reported health effects and mechanisms of egg-yolk phospholipids are illustrated in Figure 5.

## 11. Egg Phospholipids as Functional Food Ingredients

Egg yolk’s emulsifying capacity, considered here as a technological rather than nutritional property, arises from surface-active proteins and phospholipids working in concert. Whole egg yolk contains surface-active protein fractions that adsorb at the oil–water interface, while phospholipids reduce interfacial tension, acting as zwitterionic surfactants [34]. Consistent with the structural asymmetry described in Section 2, the two fractions do not contribute equally: LDL is generally considered the more important contributor to emulsifying properties, owing to its higher phospholipid-to-protein ratio, whereas granule-derived HDL/phosvitin complexes contribute more to gelation and Pickering-emulsion stabilization [35]. Mechanistically, this asymmetry follows from interfacial packing: LDL’s phospholipid-rich, low-viscosity surface monolayer adsorbs rapidly at the oil–water interface and lowers interfacial tension efficiently, whereas HDL/phosvitin’s higher protein content favors a thicker, more viscoelastic interfacial film that resists coalescence but adsorbs more slowly, favoring gel-like and Pickering-type stabilization over rapid emulsification. Phospholipase-based enzymatic modification is a well-documented lever for adjusting this functionality: PLA2-modified egg yolk shows substantially higher emulsifying capacity than PLA1-modified yolk, an effect attributed to the improved stability and emulsifying ability of the resulting 1-lysophospholipid. Notably, phospholipid activity influences droplet particle size more strongly, while protein adsorption more strongly governs emulsion stability [36], two related but mechanistically distinct contributions to overall emulsion performance.

## 12. Delivery Systems and Nutraceutical Applications

Beyond conventional emulsification, egg-yolk phospholipids have accumulated a substantial body of direct evidence in nutraceutical and food-relevant liposomal delivery, where they are compared head-to-head against the incumbent plant-derived alternative, soybean phosphatidylcholine. A 2025 study extracted phospholipid-rich fractions from both egg yolk and soybean in parallel, purified each to reference-standard quality (91.37% and 82.52% phosphatidylcholine purity, respectively), and used both to encapsulate vitamin C via microfluidic liposome production. Egg-yolk phosphatidylcholine produced smaller, more uniform particles (87 nm, PDI 0.057) than soybean phosphatidylcholine (101 nm, PDI 0.062) [37]. Because the two preparations differed in phosphatidylcholine purity, purity itself may have contributed to this difference. A separate study encapsulating egg-yolk immunoglobulin via SC-CO_2_-assisted chitosan-liposomes found the same directional result: liposomes prepared with egg-yolk phosphatidylcholine (EPC) showed better homogeneity and higher encapsulation efficiency than those prepared with soybean phosphatidylcholine (SPC) [38]. Egg-yolk lecithin, extracted via an ethanol/acetone protocol and used alone, has been formulated into liposomes entrapping the dietary flavonoid rutin, achieving entrapment efficiency up to 87% with particles under 140 nm; these rutin-liposomes attenuated glutamate-induced cytotoxicity in a human neuroblastoma cell line, pointing toward a neuroprotective application [39]. In a related formulation study, egg-yolk lecithin liposomes prepared at an optimized cholesterol-to-lecithin ratio (1:15) achieved an encapsulation efficiency of 90.92% ± 0.47% for lychee-pericarp oligomeric procyanidins, producing 80–100 nm particles that remained stable at 4 °C while measurably protecting the procyanidins’ antioxidant activity (across FRAP, ORAC, and cellular antioxidant assays) during storage [40]. Outside the food-relevant applications, liposomes made from egg-yolk lecithin have separately been shown to be less permeable, and therefore potentially better suited to controlled-release applications, than those made from soy lecithin [41], while a direct physico-chemical comparison of egg- vs. soy-lecithin liposomes found measurably different pH, viscosity, and membrane-fluidity behavior as a function of lipid concentration [42].

The formulation-specific advantages of egg-yolk phosphatidylcholine over its soybean counterpart reported in individual studies (smaller, more uniform particles in [37]; better homogeneity and higher encapsulation efficiency in [38]) may plausibly be related to compositional differences between the two sources, although the studies differ in cargo, preparation method, and outcome measures, and no single comparison establishes a general advantage. Egg-yolk phosphatidylcholine carries a higher proportion of saturated and monounsaturated acyl chains and a higher phosphatidylcholine-to-phosphatidylethanolamine ratio than soybean lecithin, which is comparatively enriched in polyunsaturated linoleic and linolenic acid chains. These differences influence acyl-chain packing density and bilayer curvature: the more saturated, PC-dominant egg-yolk profile favors tighter, more uniform bilayer packing, consistent with the smaller particle size and lower polydispersity reported for egg-yolk liposomes in [37], while the more unsaturated, PUFA-rich soybean profile produces a looser, more fluid and more permeable bilayer. This compositional rationale offers a plausible link between the egg vs. soy differences reported above and the structural premise developed in Section 2, but it should be regarded as a hypothesis rather than an established mechanism.

**Figure 6:**
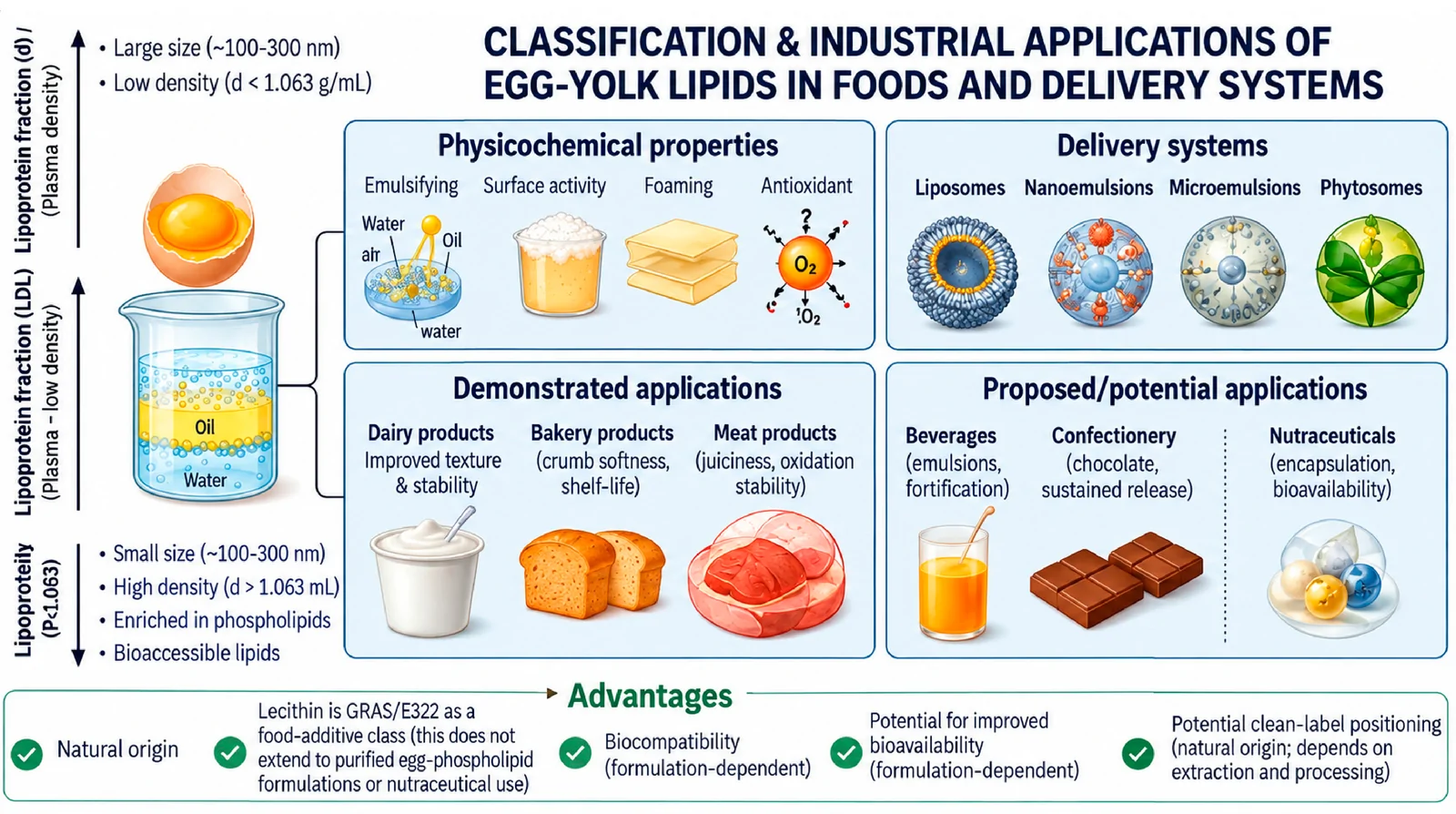
Egg phospholipids as functional ingredients and delivery systems. Evidence shown here is at the isolated liposome/ingredient level; finished-food-product incorporation with sensory or storage validation has not yet been reported (Section 12). Applications are grouped by level of support. Demonstrated applications are those for which product-specific data are discussed in this review; proposed applications (e.g., categories for which product-specific concentration, sensory, or consumer-acceptance data were not identified) are potential uses that require experimental validation. The advantages shown are potential and formulation-dependent. GRAS/E322 status applies to lecithin as a food-additive class and does not constitute validation of all purified egg-phospholipid formulations or nutraceutical uses.

This body of work is more advanced than a casual reading of the field would suggest, though it stops short of the finished product: no identified study incorporates an egg-phospholipid liposome into a finished food product with accompanying storage or sensory evaluation, and no human or in vivo bioavailability data exist for any egg-phospholipid-encapsulated nutraceutical. Both points are flagged in Section 16 as specific, actionable research priorities, distinct from a general absence of delivery-system evidence, since in vitro, formulation-level evidence favoring egg-yolk phospholipid over soy in specific systems is already available. Functional applications of egg-yolk phospholipids in foods and delivery systems are described in Figure 6.

## 13. Food Applications, Product Development, and Flavor Chemistry

### 13.1. Food Applications and Product Development

Of all the technological applications reviewed here, mayonnaise and emulsion-type sauces remain the best documented. Egg yolk (whether used whole, fractionated into LDL or granule, or enzymatically modified) is established and in active commercial or experimental use as the primary emulsifier in these products. LDL-fraction-enriched formulations, derived as co-products of industrial IgY antibody extraction, have been specifically evaluated as a mayonnaise ingredient [43], while granule-based Pickering emulsions have demonstrated the capacity to stabilize high-internal-phase emulsions with oil fractions up to 85% [35]. Baked goods, cream, and ice-cream-type products are also frequently cited as established application categories for egg-yolk lipid functionality, though this review did not identify product-specific concentration, sensory, or consumer-acceptance data for novel egg-lipid-modified formulations in these categories, a genuine gap in the applied literature rather than an oversight in this synthesis.

### 13.2. Flavor, Aroma, and Lipid-Derived Volatiles

This section is kept brief given the comprehensive coverage already provided by a 2023 review specifically addressing egg-yolk phospholipids as precursors of fatty-note odorants [3]. The core finding worth carrying forward here is that phospholipid oxidation, rather than triglyceride oxidation, is the principal source of characteristic fatty-note volatile compounds in heated egg products, with phosphatidylcholine (the dominant phospholipid class established in Section 2) correspondingly the dominant volatile precursor by mass contribution [3]. This connects mechanistically to Section 5, since the same heat-driven phospholipid degradation and lysophospholipid formation documented during spray-drying and boiling is responsible for desirable flavor development at moderate oxidation intensity and for off-flavor development once oxidation becomes excessive. Flavor chemistry and structural stability are thus best understood as the same underlying process observed from different endpoints. The applications discussed in Section 11, Section 12 and Section 13 are cataloged in Table 7.

## 14. Safety, Allergenicity, and Regulatory Considerations

Egg allergy is often treated in functional-ingredient reviews as a generic caveat, but direct experimental evidence indicates that the egg-yolk lipid fraction itself, not only co-located egg-white proteins, contributes mechanistically to allergic sensitization. In mice exposed intragastrically to egg lipids and egg lipid fractions, alone and in combination with egg-white proteins, egg triacylglycerides and phospholipids were found to differently influence the immunostimulating properties of co-administered egg-white allergens: triacylglycerides promote Th2 responses at the small-intestine level, while phospholipids reduce the solubility of egg-white proteins and induce Th2 skewing specifically in intestinal lymphoid tissue [45]. The phospholipid fraction tested in that study was 77.8% phospholipid-enriched (83.2% PC, 16.4% PE), a purity level within the range reported for several of the extraction methods summarized in Table 3, which suggests that this sensitization mechanism may be relevant at technically achievable phospholipid purities, not merely at theoretical 100%-pure preparations. A longer-term, adjuvant-free murine sensitization model reinforces the point: the whole egg lipid fraction, triacylglycerides, and phospholipids all induced allergic sensitization to egg-white proteins, with triacylglycerides favoring absorption through intestinal epithelial cells and phospholipids favoring uptake via Peyer’s patches [46].

On the clinical side, two egg-yolk allergens are of established significance: Gal d 5 (α-livetin/chicken serum albumin, heat-labile) and Gal d 6/YGP42 (a lipoprotein, heat-resistant). Egg-yolk proteins are generally less allergenic than egg-white proteins, and egg allergy affects an estimated 0.5–2.5% of young children [47]. On the regulatory side, lecithin (regardless of source) is classified in the United States as Generally Recognized As Safe (GRAS) under 21 CFR Part 184, and in the European Union as food additive E322, with no numerical Acceptable Daily Intake required following EFSA’s 2017 re-evaluation, which concluded lecithins are well tolerated, non-genotoxic, and non-carcinogenic at typical dietary exposure [48,49]. Regulatory safety clearance, however, is a distinct matter from allergen-labeling obligation: EU Regulation 1169/2011 requires the source of lecithin to be declared when derived from egg or soy, since both are designated major allergens [50], a practical commercial consideration that distinguishes egg-derived phospholipid ingredients from most plant-derived alternatives other than soy.

This regulatory picture leaves a real interpretive gap that this review considers worth naming explicitly. GRAS/E322 status reflects safety evaluation of lecithin as a food-additive class at typical dietary exposure; it does not directly address the Th2-adjuvant sensitization mechanism described above, which was characterized at the higher, more concentrated doses relevant to functional-food or nutraceutical application rather than trace additive use. This distinction is not addressed together anywhere in the literature identified for this review, and is flagged as a specific gap in Section 16.

## 15. Sustainability, Circular Bioeconomy, and Industrial Feasibility

### 15.1. Sustainability and Circular Bioeconomy

Circular bioeconomy thinking applied to livestock-derived food byproducts generally follows a “Food Waste Hierarchy” or “Value Pyramid,” in which byproducts are first recovered for direct human food use, then animal feed, then industrial (non-food) application, with bioenergy conversion occupying the lowest-priority tier [51]. Egg-yolk lipid and phospholipid recovery has been demonstrated within this hierarchy as a valorization pathway for leftover yolk material remaining after immunoglobulin Y extraction, recovering phosphatidylcholine at up to 94% from what would otherwise be a discarded processing stream [12], representing a literature-documented circular-bioeconomy application rather than merely an aspirational one. A dedicated life-cycle assessment specific to egg-yolk lipid or phospholipid extraction has not yet been reported, in contrast to the LCA methodology already applied to whole-egg production and eggshell-waste valorization. This absence follows directly from the extraction-method gap identified in Section 3, and the two limitations are best understood as connected rather than independent.

### 15.2. Industrial Feasibility and Scale-Up

The pilot-scale SFE-plus-antisolvent-precipitation process described in Section 3, which achieved 84–87% PC purity, is the most advanced scale (pilot, not merely laboratory) demonstrated specifically for egg-yolk phospholipid purification identified in this review [11]. No egg-lipid-specific techno-economic analysis was identified alongside it, which stands in clear contrast to SC-CO_2_ extraction of other lipid-bearing feedstocks (microalgae, grape seed, and various botanical extracts) where techno-economic modeling is well established and published. This contrast is drawn only to illustrate that the modeling methodology exists and is transferable in principle; it does not provide egg-lipid-specific cost data, and evidence from unrelated feedstocks should not be mistaken for direct evidence about egg-derived lipids.

A genuine industrial-feasibility tension sits underneath this picture: pharmaceutical-grade phospholipid purity is cost-prohibitive for food-industry use, which motivates interest in simpler, less-purified egg-yolk extracts that retain co-extracted vitamins and fats as a more economically viable alternative for food applications [52]. Combined with the regulatory finding from Section 14 (that egg-sourced lecithin carries the same GRAS/E322 status as soy or sunflower lecithin, but a mandatory allergen-labeling requirement that sunflower lecithin does not carry), the overall industrial-feasibility picture that emerges is a mixed one: technologically mature at pilot scale for purification, safety-cleared as a food-additive class, but lacking dedicated techno-economic validation and, at present, any clear cost or labeling advantage over established plant-derived alternatives.

## 16. Knowledge Gaps and Research Limitations

Eight gaps recur across the sections above, and each is stated here with the specific study design that would close it.

1. Whole-egg vs. isolated egg-phospholipid-ingredient bioavailability. Human intervention data exist for isolated egg-yolk-phospholipid choline bioavailability (an egg-phospholipid drink, not whole egg) [19] and for whole-egg cholesterol absorption [21], but no identified human study directly compares whole-egg consumption against an isolated or purified egg-phospholipid ingredient for the same outcome—indeed, the only whole-egg choline trial identified in this review [20] compared eggs with choline bitartrate rather than with an isolated egg-phospholipid ingredient, which itself illustrates the gap. This is one of the two most consequential gaps for functional-ingredient translation identified in this review; a three-arm randomized crossover trial (whole egg vs. isolated egg-PL ingredient vs. non-egg control) measuring plasma choline and/or lipid biomarker AUC would resolve it.
2. Standardized reference values for egg-lipid composition. Reported phospholipid-class percentages vary meaningfully across studies (PC reported at 72–77% of total phospholipid across independent sources), which confounds cross-study synthesis; an interlaboratory ring trial applying a single standardized protocol across hen breeds and diets is required.
3. Functional role of newly resolved minor lipid species. Recent lipidomics identified 19 phospholipid classes and 275 molecular species, including previously uncharacterized glucosylceramides, gangliosides, and PUFA-carrying plasmalogens [7], whose bioactivity remains untested; targeted in vitro bioactivity screening of isolated fractions is needed.
4. Life-cycle and techno-economic data for egg-lipid extraction. No dedicated LCA or techno-economic analysis specific to egg-yolk lipid extraction was identified, despite mature modeling methodology existing for adjacent feedstocks; applying established SC-CO_2_ techno-economic modeling to egg-yolk phospholipid extraction at pilot-to-industrial scale is required.
5. Dose-escalated safety and allergenicity-mechanism integration. GRAS/E322 status covers trace additive-level exposure but does not address the documented Th2-adjuvant sensitization mechanism at functional-ingredient-relevant concentrations [45,46]; dose–response allergenicity/immunogenicity testing of a purified egg-phospholipid ingredient is required.
6. Egg-specific oral/food nutraceutical delivery validation. Egg-yolk lecithin has demonstrated strong encapsulation efficiency for a food-relevant polyphenol in isolated liposome form [39,40], but no identified study incorporates an egg-phospholipid liposome into a finished food product with storage/sensory evaluation, and no human or in vivo bioavailability data exist for any egg-phospholipid-encapsulated nutraceutical.
7. HDL vs. LDL fraction digestion kinetics. Egg-yolk HDL and LDL fractions produce divergent metabolic effects in animal models [23], yet no study has directly compared their digestion kinetics under standardized conditions. Parallel INFOGEST digestion of isolated HDL and LDL fractions with lipidomic monitoring would address this.
8. Egg-specific cognitive/neurological human evidence. The existing human RCT evidence for phospholipid-linked cognitive benefit used marine-sourced PS-DHA rather than egg-derived phospholipid [33]. A human RCT of isolated egg-derived phosphatidylserine or phosphatidylcholine on validated cognitive outcomes would be needed to establish or refute egg-specific bioactivity that is currently inferred only by analogy. These eight gaps, together with the study design required to close each one, are set out in Table 8.

## 17. Future Research Agenda

The eight gaps above do not carry equal urgency. The time-horizon groupings that follow reflect the authors’ own assessment and recommendation for where effort might reasonably go first, rather than a ranking derived quantitatively from the literature itself.

In the short term (1–3 years), the priorities are largely methodological and mechanistic: standardized analytical methods and lipidomic reference ranges (Gap 2) via interlaboratory ring trials; targeted bioactivity screening of the newly resolved ganglioside and plasmalogen fractions (Gap 3); a standardized digestion comparison of isolated HDL vs. LDL fractions (Gap 7); and dose–response allergenicity testing of a purified egg-phospholipid ingredient (Gap 5). None of these require new extraction technology to be developed first; they can proceed with methods already established elsewhere in this review.

In the medium term (3–5 years), the field’s highest-priority human study becomes feasible: a three-arm human bioavailability trial comparing whole egg, an isolated egg-phospholipid ingredient, and a non-egg control (Gap 1). Running alongside it should be techno-economic modeling of egg-lipid extraction at pilot-to-industrial scale (Gap 4), and the first component of Gap 6: incorporation of egg-PL liposomes into a model food product with defined storage and sensory evaluation.

**Figure 7:**
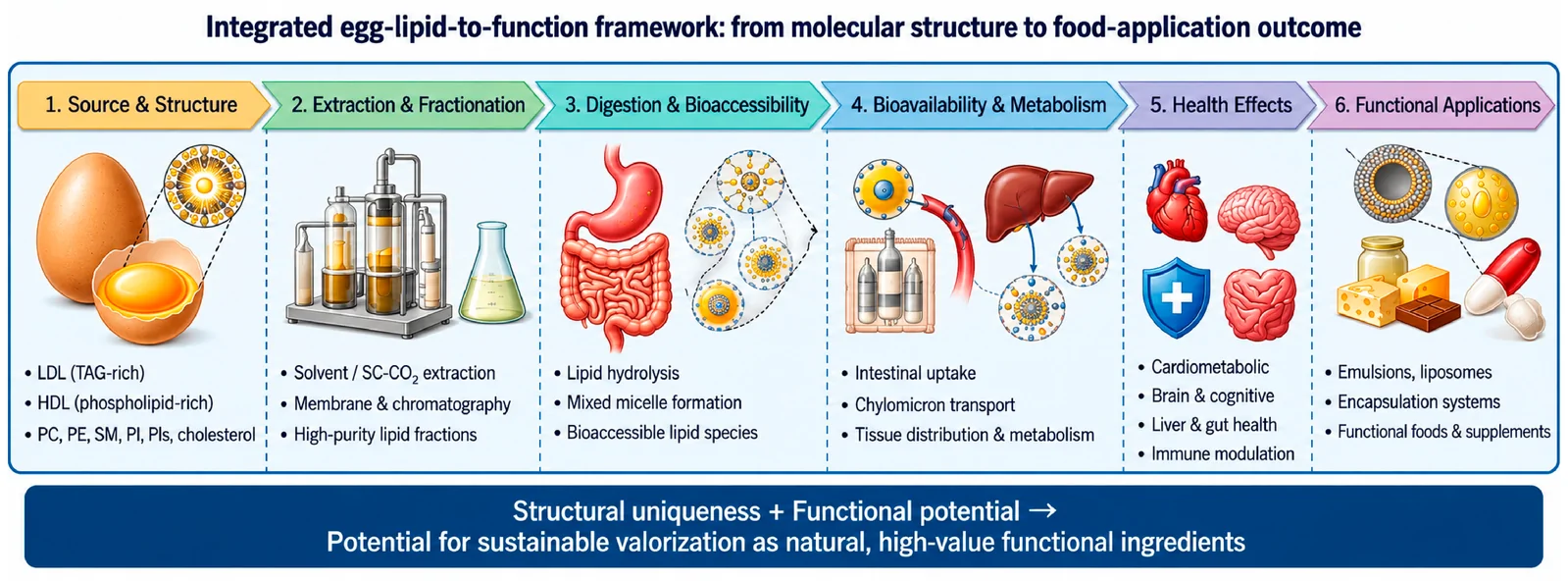
Integrated Egg-Lipid-to-Function Framework: from molecular structure to food-application outcome. Note: the food-application stage of this framework reflects technological potential; product-level sensory and consumer-acceptance validation remains an identified gap (Section 16, Gap 6). Egg-specific life-cycle assessment and techno-economic analyses are not currently available; sustainability and economic aspects are therefore presented as potential, and functional performance is formulation-dependent.

In the longer term (5–10 years), two further steps depend on the medium-term work landing first: human RCTs of isolated egg-derived phosphatidylserine and phosphatidylcholine on cognitive and metabolic outcomes, resolved separately from the marine-PS evidence (Gap 8); and pilot human bioavailability studies of egg-phospholipid-encapsulated nutraceuticals in finished food products (Gap 6, second component). A dedicated life-cycle assessment should feed into commercial-scale biorefinery design over this same horizon, one that integrates the demonstrated IgY-byproduct valorization pathway as a template applicable to other egg-processing co-product streams. Integrated Egg-Lipid-to-Function Framework is clarified in Figure 7.

## 18. Conclusions

Egg-derived lipids and phospholipids are structurally distinct, compositionally heterogeneous, and compartmentalized between two functionally divergent lipoprotein pools; the consequences for digestion are inferred mechanistically from lipoprotein structure; direct experimental comparison of the digestion kinetics of isolated HDL and LDL fractions has not yet been performed and remains an important target for future work. The strongest human evidence available, which is limited to a single small crossover trial for choline and to postprandial plasma responses for cholesterol, suggests that phospholipid-bound choline may be more bioavailable than synthetic choline salts and that the whole-egg matrix may limit the postprandial appearance of cholesterol: evidence that directly informs, but does not settle, whether these advantages persist once the phospholipid is isolated and purified into a standalone functional ingredient. Extraction and delivery-system technology for egg-derived phospholipids is, in fact, more mature than commonly assumed, encompassing genuine pilot-scale purification and formulation-specific egg vs. soy comparisons in liposomal delivery. This technological maturity, however, has not yet translated into finished, sensory-validated food products; product-level and consumer-acceptance evidence remains a distinct and unresolved limitation, separate from the ingredient- and delivery-system-level evidence reviewed above. What remains is narrower than a general evidence shortfall: techno-economic and life-cycle validation, and a direct whole-egg vs. isolated-ingredient human bioavailability comparison, stand as the two most consequential and most specifically actionable gaps between egg-derived lipids as documented bioactive compounds and egg-derived lipids as validated, commercially deployed functional food ingredients.

## Figures and Tables

**Table 1:** Major egg-yolk lipid classes: composition, structural location, and reported abundance.

Lipid Class	Reported Abundance	Structural Location	Source
Triacylglycerols (TAG)	62–70% of total yolk lipid	LDL core (dominant); minor in HDL	[5,6]
Total phospholipids	25–33% of total yolk lipid	HDL/granule surface (enriched); LDL surface monolayer	[5,6]
Cholesterol (free + esterified)	4–5% of total yolk lipid	LDL core (esters) and particle surface (free)	[5,6]
LDL particle lipid	83–89 wt% of particle; ~69% TAG/26% PL/5% cholesterol	Plasma fraction (~85% of plasma)	[6]
Minor lipid-associated compounds (carotenoids, fat-soluble vitamins)	Not reported (diet-dependent)	Associated with LDL/plasma	[2]

**Table 2:** Egg phospholipid classes and reported composition.

Phospholipid Class	Reported % of Total PL	Notable Molecular Species	Source
Phosphatidylcholine (PC)	72–77%	PC (16:0–18:1)	[3,6]
Phosphatidylethanolamine (PE)	15–20%	PE (18:0–20:4); DHA often at sn-2	[3,6,7]
Sphingomyelin (SM)	~3%	SM (d18:1/16:0)	[6]
Lysophosphatidylcholine (LPC)	~3%	LPC (16:0)	[6]
Phosphatidylinositol (PI)	~2%	PI (18:0–18:2)	[6,7]
Phosphatidylserine (PS)	~1.3%	PS (18:0–18:2)	[3,6]
Plasmalogens, gangliosides, glucosylceramides	Not reported quantitatively	PUFA-enriched at sn-2; newly resolved	[7]
Total resolved classes/subclasses (lipidomics)	19 classes/275 molecular species	Reflects LC-MS/MS depth, not classical panel	[7]

**Table 3:** Extraction and fractionation technologies applied to egg-yolk lipids: yield, purity, advantages, and limitations.

Method	Yield/Recovery	Purity	Key Limitation	Source
Bligh and Dyer (solvent)	63 g/100 g sample	29% PL in extract	Toxic chloroform; under-recovers acidic PLs	[10]
SC-CO_2_, no modifier	67 g/100 g sample	26% PL in extract	Limited PL solubility in neat CO_2_	[10]
Pilot-scale SFE + antisolvent precipitation	Not reported as single figure	PC purity 84–87%	Purity varies strongly with feed moisture	[11]
SC-CO_2_ + ethanol, leftover yolk (post-IgY)	PC recovery 40–94% (fraction-dependent)	PC purity 47–75%	Recovery and purity trade off	[12]
Enzymatic (protease) pretreatment + SC-CO_2_	PL recovery 86% (vs. 49% untreated)	PL purity 67% (reduced)	Co-extracted peptides reduce purity	[12]
Subcritical propane extraction (SPE)	Highest overall efficiency of 3 methods	Intermediate PL extraction	Not phospholipid-selective	[13]
Switchable-hydrophilicity solvents/NADES	Not reported for egg specifically	Not reported for egg specifically	Egg-specific validation not yet reported	[9]

**Table 4:** Analytical techniques for egg-lipid characterization.

Technique	What It Measures	Key Limitation	Source
TLC	Class presence/absence	Low resolution; laborious quantitation	[9]
UHPLC/HPLC-CAD/ELSD	Class-level quantification	Co-elution of minor lyso-species	[9]
LC-ESI-MS(/MS)	Molecular-species ID; oxidation/lyso-PL tracking	Ion suppression without clean input	[7,9]
^31^P NMR	Non-destructive, quantitative class profiling	Lower sensitivity; high instrument cost	[9]
GC-FAME	Fatty-acyl composition of separated fractions	Cannot resolve intact phospholipids	[9]
Untargeted lipidomics (LC-MS/MS, high-res)	Full lipidome incl. minor/novel classes	Requires specialized expertise; costly	[7]

**Table 5:** Digestion, bioaccessibility, absorption, and metabolic fate of egg-derived lipids.

Stage/Parameter	Finding	Evidence Level	Source
Lipid bioaccessibility vs. thermal processing (65–85 °C)	FFA bioaccessibility 25–29% (raw) vs. 20–24% (85 °C)	In vitro (TIM-1)	[16]
HHP effect on granule digestion (INFOGEST 2.0)	Increased PL detection at oral/gastric phase; no change to protein digestibility overall	In vitro (INFOGEST)	[17]
Biopolymer encapsulation, digestion rate	Reduced lipid/cholesterol digestion rate; lower FFA and oxidation	In vitro (human digestion model)	[18]
Choline bioavailability, egg-PL vs. bitartrate	4x higher plasma choline AUC	Human intervention RCT (*n* = 18)	[19]
Choline bioavailability, metabolic syndrome cohort	Both sources raised plasma choline; no TMAO change	Human intervention RCT	[20]
Postprandial plasma cholesterol AUC, whole-egg matrix	No significant change in plasma cholesterol AUC	Human intervention (2 RCTs)	[21]
PC-cholesterol transporter mechanism	Proposed interaction with NPC1L1/SR-B1	Mechanistic hypothesis	[22]
Whole egg vs. isolated PL ingredient, direct comparison	Not reported, not identified in the literature		Gap

**Table 6:** Evidence for biological and metabolic effects of egg-derived lipids.

Claimed Effect	Finding	Evidence Level	Source
Hepatic lipid partitioning (LDL vs. HDL)	LDL raises liver/plasma cholesterol; HDL lowers body/epididymal fat	Animal (mouse)	[23]
Hepatic lipogenic gene suppression	SREBP-1c down 3.9–9.4-fold; Elovl6 down	Animal (mouse)	[24]
Hepatic glycerolipid secretion shift	Increased PL secretion, decreased TG secretion	Animal (isotope tracer)	[25]
Anti-inflammatory/hypolipidemic, egg vs. soy PL	Both reduce hepatic TG/oxidative markers; egg PL raises acetylcholine	Animal (rat)	[27]
All-cause/cancer mortality dose–response	+7%/day all-cause, +13%/day cancer; no CVD mortality link	Human meta-analysis (55 cohorts)	[28]
CVD incidence, large cohorts	HR 0.93 (95% CI 0.82–1.05), no significant association	Human observational (3 cohorts)	[29]
Postprandial plasma cholesterol AUC, whole egg	No significant plasma cholesterol AUC change	Human intervention (2 RCTs)	[21]
Cognitive effect, PS-DHA	Improved verbal recall in elderly	Human RCT, marine PS, not egg-derived	[33]
Choline bioavailability	4x higher plasma AUC vs. bitartrate	Human intervention RCT	[19]

**Table 7:** Food and delivery-system applications: technological functionality and evidence status.

Application	Lipid/Fraction Used	Function	Evidence Status	Source
Mayonnaise/salad dressing	Whole yolk, LDL, granules, PLA2-modified yolk	Primary emulsification	Established	[43,44]
High-internal-phase Pickering emulsions	Egg-yolk granules	Stabilizes up to 85% oil fraction	Established (lab/pilot)	[35]
IgY-loaded chitosan-liposome (SC-CO_2_)	Egg PC (EPC) vs. soy PC (SPC)	EPC: better homogeneity, higher EE	Established (lab)	[38]
Polyphenol (rutin) liposome, neuroprotective	Egg-yolk lecithin only	87% entrapment; attenuated glutamate cytotoxicity (SH-SY5Y)	Established (lab), egg-specific	[39]
Procyanidin (OPC) liposome, antioxidant/storage	Egg-yolk lecithin only	90.92% encapsulation efficiency; preserved antioxidant activity	Established (lab), egg-specific	[40]
Cosmetic/topical liposomes	Egg vs. soy lecithin	Lower permeability (egg) vs. soy; differential pH/viscosity behavior	Established (lab, non-food)	[41,42]
Food-matrix incorporation with storage/sensory data	-	-	Not reported—gap	-
Human/in vivo bioavailability of encapsulated nutraceutical	-	-	Not reported—gap	-
Flavor/aroma (fatty-note volatiles)	Phosphatidylcholine (dominant)	Principal precursor under heat/oxidation	Established (competing review)	[3]

**Table 8:** Knowledge gaps, translational barriers, and future research priorities.

Research Gap	Current Problem	Required Research	Priority
Whole-egg vs. isolated ingredient bioavailability	No direct human comparison identified	3-arm RCT: whole egg vs. isolated PL ingredient vs. control	Medium-term
Standardized compositional reference values	PC reported 72–77% across studies	Interlaboratory ^31^P NMR ring trial	Short-term
Functional role of minor lipid species	19 PL classes/275 subclasses identified; function untested	Targeted in vitro bioactivity screening	Short-term
Life-cycle/techno-economic data for extraction	No egg-specific LCA or TEA identified	Apply established SC-CO_2_ TEA methodology	Medium-term
Dose-escalated safety + allergenicity mechanism	GRAS covers trace additive use only	Dose–response allergenicity study	Short-term
Egg-specific oral delivery validation	Strong in vitro EE%; no in-product/in vivo data	Food-matrix incorporation + pilot human study	Medium/Long-term
HDL vs. LDL digestion kinetics	Divergent metabolic effects; digestion origin untested	Parallel INFOGEST digestion with lipidomics	Short-term
Egg-specific cognitive/neurological evidence	Existing RCT evidence is marine-PS, not egg-PS	Human RCT of isolated egg-PS/PC on cognition	Long-term

## Data Availability

No new data were created or analyzed in this study. Data sharing is not applicable to this article.
